# The role of indoleamine 2,3-dioxygenase-aryl hydrocarbon receptor pathway in the TLR4-induced tolerogenic phenotype in human DCs

**DOI:** 10.1038/srep43337

**Published:** 2017-03-03

**Authors:** Fabián Salazar, Dennis Awuah, Ola H. Negm, Farouk Shakib, Amir M. Ghaemmaghami

**Affiliations:** 1 Division of Immunology, School of Life Sciences, Faculty of Medicine and Health Sciences, University of Nottingham, United Kingdom; 2 Medical Microbiology and Immunology Department, Mansoura University, Egypt

## Abstract

A controlled inflammatory response is required for protection against infection, but persistent inflammation causes tissue damage. Dendritic cells (DCs) have a unique capacity to promote both inflammatory and anti-inflammatory processes. One key mechanism involved in DC-mediated immunosuppression is the expression of tryptophan-metabolizing enzyme indoleamine 2,3-dioxygenase (IDO). IDO has been implicated in diverse processes in health and disease but its role in endotoxin tolerance in human DCs is still controversial. Here we investigated the role of IDO in shaping DCs phenotype and function under endotoxin tolerance conditions. Our data show that TLR4 ligation in LPS-primed DCs, induced higher levels of both IDO isoforms together with the transcription factor aryl-hydrocarbon receptor (AhR), compared to unprimed controls. Additionally, LPS conditioning induced an anti-inflammatory phenotype in DCs - with an increase in IL-10 and higher expression of programmed death ligand (PD-L)1 and PD-L2 - which were partially dependent on IDO. Furthermore, we demonstrated that the AhR-IDO pathway was responsible for the preferential activation of non-canonical NF-κB pathway in LPS-conditioned DCs. These data provide new insight into the mechanisms of the TLR4-induced tolerogenic phenotype in human DCs, which can help the better understanding of processes involved in induction and resolution of chronic inflammation and tolerance.

Inflammation is a complex pathophysiological state orchestrated primarily by innate immune cells in response to infection and/or tissue damage. This phenomenon is indispensable for protecting the host from invading pathogens. However, overproduction of inflammatory molecules can cause tissue damage and has been implicated in the onset of pathological hyper-inflammatory responses, including allergy, atherosclerosis, sepsis and a number of autoimmune diseases^1^,^2^. Innate immune cells have developed mechanisms to counteract over-inflammatory responses and reduce their negative effect by restoring homeostasis. Indoleamine 2,3-dioxygenase (IDO) is an immune-regulatory enzyme, mainly expressed in dendritic cells (DCs), that can modulate adaptive immune responses by promoting immune-suppression and tolerance^3^,^4^.

Under inflammatory conditions, IFN-γ can induce high levels of IDO which, through tryptophan (TRP) depletion and production of TRP metabolites can help to resolve inflammation and restore tissue homeostasis^3^,^4^. Additionally as we and others have shown, there is an IFN-γ-independent pathway for induction of IDO which is mainly mediated by toll-like receptors (TLRs) such as TLR4 in myeloid DCs^5^,^6^,^7^ and TLR9 in plasmacytoid DCs^8^,^9^. This up-regulation of IDO by TLR ligands involves pathways that converge in non-canonical nuclear factor-kappaB (NF-κB)^7^,^8^,^9^. Interestingly, the ligand-dependent transcription factor aryl-hydrocarbon receptor (AhR) - originally implicated in the mechanisms behind the toxicity mediated by xenobiotics - has been closely linked with IDO in modulation of immune responses^10^. Particularly, it has been shown that an interaction between kynurenine (KYN), the main metabolite produced in the IDO-mediated TRP-catabolizing pathway, and AhR can modulate DC immunogenicity and induce regulatory T cells^11^,^12^. More recently, the AhR-IDO axis has been shown to be involved in endotoxin tolerance in a mouse model^13^.

DCs are professional antigen presenting cells and key regulators of adaptive immunity. They possess high functional plasticity in response to external stimuli, which allows them to orchestrate adaptive immune responses by either promoting or suppressing T-cell responses. Antigen recognition and uptake in the periphery followed by migration to secondary lymphoid organs along with expression of co-stimulatory molecules and secretion of cytokines are the main features that enable DCs to stimulate naïve T cells or under specific micro-environmental conditions induce tolerance^14^,^15^. Lipopolysaccharide (LPS) activates the TLR4 pathway that induces the release of pro-inflammatory cytokines and the induction of co-stimulatory molecules necessary to activate immune responses. Engagement of TLR4 and specific signaling adaptor molecules such as MyD88 and MyD88-adaptor-like protein (MAL) or TRIF and TRIF-related adaptor molecule (TRAM) stimulate downstream signaling pathways that ultimately lead to the activation of different mainly pro-inflammatory transcription factors including NF-κB pathway^16^. Interestingly, LPS has also been shown to induce tolerance after repeated stimulation leading to immune-suppression but the mechanism of such tolerance is still not fully understood^17^,^18^,^19^,^20^.

Here we studied the effect of LPS-conditioning on the expression of immuno-modulatory molecules IDO and AhR in human DCs. We also investigated the role of these molecules in modulating DC phenotype and function under endotoxin tolerance conditions. Additionally, we studied the link between the IDO and the canonical and non-canonical NF-κB pathways under these conditions in order to better understand their role in TLR4-mediated IDO modulation and endotoxin tolerance in human DCs.

## Results

### LPS priming induces the IDO pathway in human DCs

**Figure 1:**
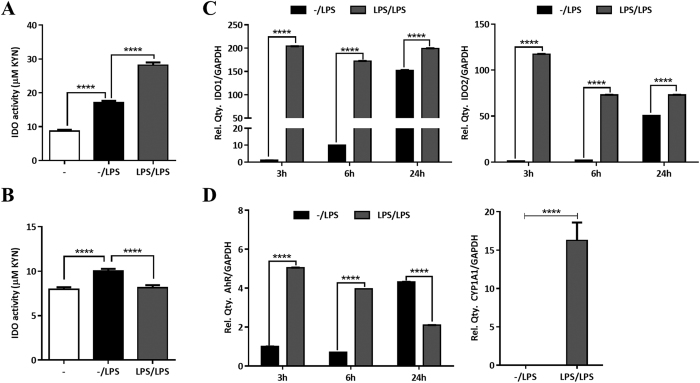
The IDO pathway is induced in LPS-conditioned human DCs but not monocytes. (**A**) IDO activity in human DCs stimulated either once (unprimed DCs) or twice (LPS-primed DCs) with LPS (0.01 μg/ml) for 24 hrs (n = 6). (**B**) IDO activity in human monocytes stimulated either once or twice with LPS (0.01 μg/ml) for 24 hrs (n = 4). (**C**) qRT-PCR analysis of IDO1 and IDO2 gene expression in unprimed versus LPS-primed DCs after re-stimulation with LPS. (One representative experiment out of three). (**D**) qRT-PCR analysis of AhR (3, 6, 24 hrs) and CYP1A1 (24 hrs) gene expression in unprimed versus LPS-primed DCs after re-stimulation with LPS (one representative experiment out of three). Relative expression of IDO1, IDO2, AhR and CYP1A1 were compared with that of GAPDH.

It has been previously shown that TLR4 signaling can induce IDO in human DCs^5^. In particular, we have recently shown that both IDO1 and IDO2 are induced after single stimulation with LPS in human DCs^7^. In this study, we aimed to investigate IDO regulation after TLR4 ligation in LPS-primed human DCs as an *in-vitro* model of endotoxin tolerance. We evaluated IDO activity as well as IDO1 and IDO2 expression in human DCs that were primed with LPS compared with DCs stimulated only once with LPS (unprimed controls). As expected IDO activity was significantly induced in unprimed DCs after LPS stimulation compared with unstimulated DCs (media only). Moreover, we found a significant induction in IDO activity in LPS conditioned DCs compared with unstimulated or single LPS stimulation controls (Fig. 1A). Intriguingly, unlike DCs, monocytes did not produce high levels of IDO after subsequent stimulation with LPS showing that, this is a particular feature of DCs (Fig. 1B).

We then evaluated the contribution of both IDO isozymes namely IDO1 and IDO2 in IDO activity by assessing their gene expression. Our data show that single stimulation with LPS induces IDO1 as well as IDO2 gene expression in human DCs in a time-dependent manner with a peak at 24 hrs. Conversely, LPS-primed DCs produced much higher levels of both IDO isoforms at early time points (3 hrs) (Fig. 1C). Interestingly, no induction in IDO2 was reported in mouse splenic DCs under similar conditions^21^, underlying species specific characteristics in terms of IDO regulation.

The ligand-dependent transcription factor AhR has been shown to mediate some of the immuno-modulatory functions of IDO metabolites and regulate IDO1 signaling in mouse DCs^11^,^12^,^13^. Here our data show that upon LPS stimulation AhR gene expression and activity, assessed by measuring the expression of one of its target genes named cytochrome P450 family 1 member 1 (CYP1A1), are highly induced in LPS-conditioned DCs compared with unprimed controls. The kinetics of AhR induction was similar to the one observed for IDO1 and IDO2, with high levels after 24 hrs in unprimed controls and early induction at 3 hrs in LPS-primed DCs (Fig. 1D). These data underline the potential role of the AhR as well as both IDO isoforms in the induction of endotoxin tolerance in human DCs.

### LPS conditioning induces an anti-inflammatory phenotype in human DCs

**Figure 2:**
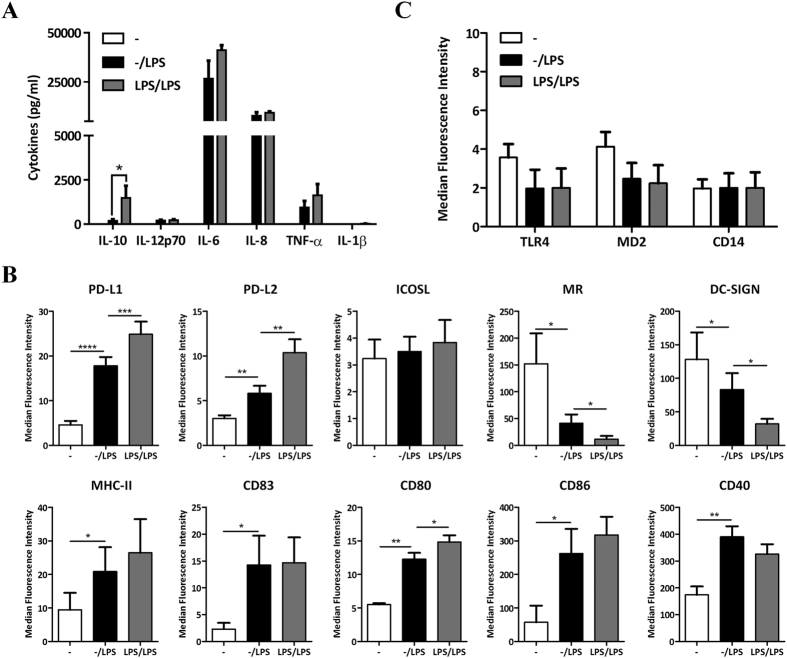
LPS-conditioning induce an anti-inflammatory phenotype in DCs. (**A**) Cytokine production by unprimed and LPS-primed DCs after re-stimulation with LPS (n = 5). (**B**) Flow cytometry analysis of surface protein phenotypical markers in unprimed and LPS-primed DCs after re-stimulation with LPS (n ≥ 3). (**C**) Flow cytometry analysis of TLR4, MD2 and CD14 protein expression in unprimed versus LPS-primed DCs after re-stimulation with LPS (n = 3).

One of the main features of LPS conditioned cells is their ability to differentially modulate the production of pro and anti-inflammatory cytokines after re-stimulation with LPS^17^,^18^,^19^,^20^. Accordingly, we evaluated the levels of pro-inflammatory cytokines such as IL-12p70, IL-6, IL-8, TNF-α and IL-1β, as well as the anti-inflammatory cytokine IL-10 in human LPS-primed DCs compared with unprimed controls. A significant induction in most of the cytokines tested including IL-10, IL-6, IL-8, IL-12p70 and TNF-α was observed in DCs stimulated with LPS once with no differences in the levels of IL-1β, a cytokine mainly produced by macrophages as an inactive precursor that requires activated caspase-1 to generate its biological active compound^22^. Interestingly, no significant differences were observed between LPS-conditioned and unprimed DCs in all pro-inflammatory cytokines tested. However, a significant induction in IL-10 was observed in LPS-conditioned DCs compared with single stimulated controls (Fig. 2A) after LPS stimulation.

We also studied how DC surface phenotype was affected under these conditions. Our data show a significant induction in the surface expression of inhibitory molecules including programmed death ligand (PD-L)1 and PD-L2 in LPS conditioned DCs compared to single stimulated controls. No differences were found in co-stimulatory molecules including CD40, MHC-II, CD83 and CD86. However, a slight induction in CD80 was detected (Fig. 2B). In addition, we found a significant reduction in the expression of the C-type lectin receptors DC-SIGN and mannose receptor (MR) (Fig. 2B) - which are receptors involved in antigen recognition and uptake^15^ - suggesting a decrease in the endocytic capacity of these cells. Interestingly, we did not find significant differences in the LPS receptor complex i.e. TLR4, MD2 and CD14 protein expression (Fig. 2C). All these data show that LPS-primed human DCs adopt an anti-inflammatory phenotype characterized by an increased expression in immuno-regulatory mediators such as IDO1, IDO2, AhR and IL-10, as well as the inhibitory molecules PD-L1 and PD-L2.

### IDO activity partially regulates the anti-inflammatory phenotype under endotoxin tolerance conditions

**Figure 3:**
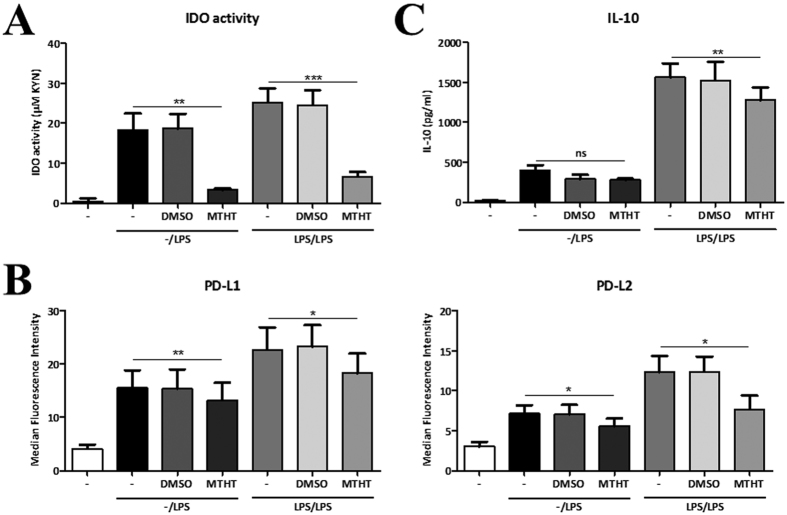
IDO partially mediates the anti-inflammatory phenotype in LPS-conditioned DCs. (**A**) IDO activity in human DCs stimulated either once or twice with LPS in the presence and absence of the IDO inhibitor MTHT (n = 3). (**B**) Flow cytometry analysis of PD-L1 and PD-L2 protein expression in unprimed versus LPS-primed DCs after re-stimulation with LPS in the presence and absence of MTHT (n = 3). (**C**) IL-10 production by unprimed and LPS-primed DCs after re-stimulation with LPS in the presence and absence of MTHT (n = 3).

We then investigated the involvement of the IDO pathway in the induction of the anti-inflammatory mediators in human DCs under endotoxin tolerance conditions. For these experiments, we stimulated LPS-primed and unprimed DCs with a subsequent dose of LPS in the presence of the IDO inhibitor methyl-thiohydantoin tryptophan (MTHT). IDO activity was substantially reduce in DCs exposed to MTHT (Fig. 3A). We also found that the presence of MTHT significantly reduces PD-L1 and PD-L2 surface expression in LPS-primed DCs as well as unprimed controls (Fig. 3B). Furthermore, IL-10 was also reduced in DCs consecutively exposed to LPS in the presence of MHT compared to DCs culture in the absence of MTHT (Fig. 3C). These data suggest a key role of the IDO pathway in modulating DC phenotype and function under endotoxin tolerance conditions.

### LPS conditioning favors the non-canonical NF-κB pathway in human DCs

**Figure 4:**
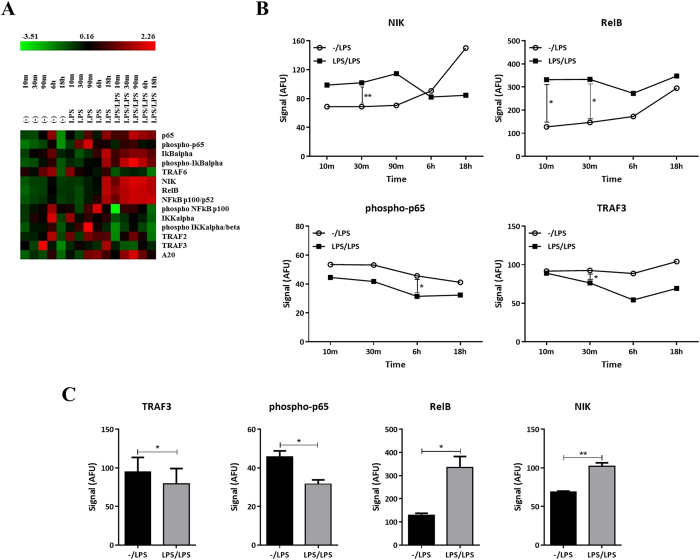
LPS-conditioning favors the non-canonical NF-κB pathway in human DCs. (**A**) Heat maps representing the relative abundance of proteins upstream the NF-κB signalling pathway using unprimed and LPS-primed DCs re-stimulated with LPS for different time points. (**B**) Protein expression of phospho-p65, NIK, RelB and TRAF3 in unprimed and LPS-primed DCs re-stimulated with LPS. Data are shown as geometric mean of three independent experiments. **(C**) Protein expression of RelB, NIK, phospho-p65 and TRAF3 in unprimed and LPS-primed DCs re-stimulated with LPS (n = 3).

The NF-κB pathway has been previously linked with the induction of endotoxin tolerance^17^,^20^,^23^ as well as IDO modulation^24^,^25^. Therefore, we studied the levels of several components of the NF-κB pathway in an attempt to understand the molecular mechanisms behind IDO modulation in LPS-conditioned DCs. We used a reverse phase protein array approach^7^,^26^ to evaluate simultaneously protein expression as well as post-translational modifications in LPS-conditioned DCs using unprimed DCs as controls. We evaluated a panel of 14 different targets including members of the NF-κB family, inhibitor of kappaB (IκB) family, IκB kinase (IKK) family, TRAF protein family as well as NF-κB regulators using β-actin as a housekeeping control. Green (low expression) to red (high expression) heat maps represent the relative abundance of proteins upstream of the canonical and non-canonical NF-κB signalling pathway (Fig. 4A). Upon LPS stimulation, we found a significant increase in the expression of key components of the non-canonical NF-κB pathway (i.e. RelB and the NF-κB-inducing kinase (NIK)) at early time points (30 min) in LPS-primed DCs compared with unprimed controls (Fig. 4B,C). Additionally, we observed a significant reduction in the activity of the canonical pathway in LPS-conditioned DCs compared with single stimulated controls. This was reflected in the levels of p65 phosphorylation at late time points (6 hrs) (Fig. 4B,C). Furthermore, our data show a significant down-regulation in the levels of TRAF3, a negative regulator of the non-canonical pathway (Fig. 4B,C). Collectively these data show that DCs stimulated once with LPS favor the canonical NF-κB pathway, as evidenced by an increase in phospho-p65 at early time points, while an induction in the non-canonical pathway was observed at late time points. Conversely, LPS-conditioning biased the activation in favor of the non-canonical pathway, demonstrated by an increase in NIK and RelB as well as a decrease in TRAF3 protein levels. This highlights the potential role of the NF-κB pathway in mediating LPS-induced tolerogenic phenotype in human DCs through IDO.

### The IDO pathway modulates RelB under endotoxin tolerance conditions

**Figure 5:**
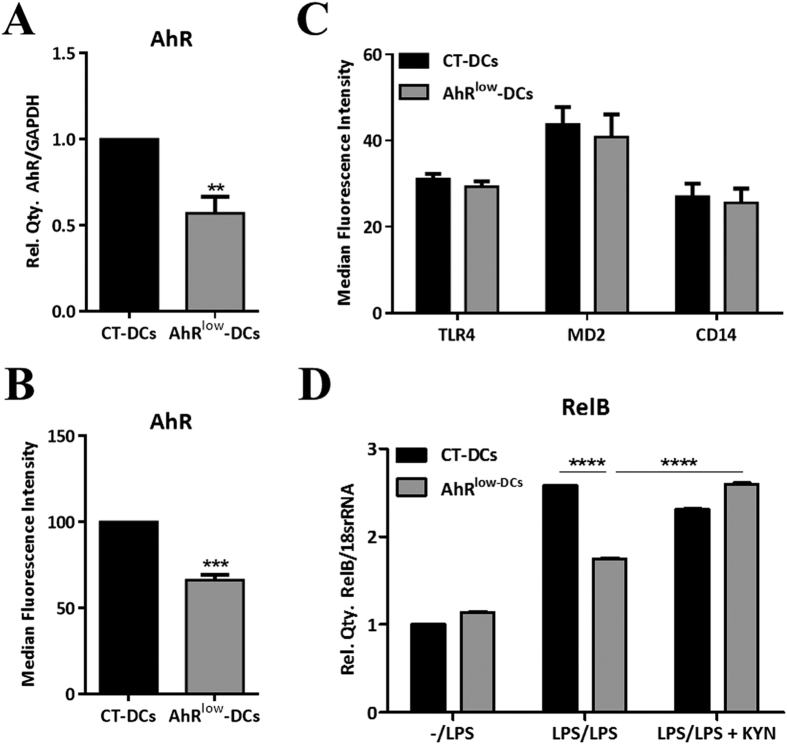
RelB is regulated by the IDO pathway in LPS-conditioned DCs. (**A**) qRT-PCR analysis of AhR gene expression in control (CT) and AhR^low^-DCs. Relative expression of AhR was compared with that of GAPDH (n = 2). (**B**) Flow cytometry analysis of AhR protein expression in CT and AhR^low^-DCs (n = 3). (**C**) Flow cytometry analysis of TLR4, MD2 and CD14 protein expression in CT and AhR^low^-DCs (n = 3). (**D**) qRT-PCR analysis of RelB gene expression in unprimed and LPS-primed DCs re-stimulated with LPS in the presence and absence of the IDO metabolite KYN. Relative expression of RelB was compared with that of 18S rRNA (one representative experiment out of two).

We showed that one of the main molecular features of LPS conditioning was the induction of the NF-κB pathway member RelB. Previously, we and others have suggested that RelB might interact with AhR in the modulation of IDO levels^7^,^27^. Accordingly, we further studied the link between the IDO pathway and RelB by generating AhR low expressing DCs (AhR^low^-DCs) using gene silencing (Fig. 5A,B). AhR^low^-DCs expressed low levels of AhR at gene and protein levels compared to control DCs (CT-DCs) (Fig. 5A,B), without affecting the expression of TLR4, MD2 and CD14, the molecules that are part of the LPS receptor complex (Fig. 5C). We then analysed RelB expression under endotoxin tolerance conditions in control (CT) and AhR^low^-DCs. Our data show that RelB is significantly reduced in LPS-conditioned AhR^low^-DCs compared to CT-DCs (Fig. 5D). Moreover, we found that this reduction in RelB was reverted in the presence of the IDO metabolite KYN (Fig. 5D). These data demonstrated the key role of the IDO pathway in the modulation of RelB under endotoxin tolerance conditions, which can help to understand how these molecules can regulate the balance between inflammation and tolerance in human DCs.

## Discussion

Uncontrolled or sustained inflammation has been implicated in the pathogenesis of many diseases including metabolic disorders, allergy, neurodegenerative diseases and cancer^28^,^29^,^30^. Despite substantive efforts, no effective treatments have been developed to combat systemic inflammatory responses such as sepsis^31^. Therefore, better understanding of the mechanisms of inflammation and immune-suppression are essential for developing more effective therapeutic strategies. Previous studies have shown that degradation of TRP and high IDO activity are associated with the development of sepsis in bacteremic patients^32^,^33^,^34^. In this context, IDO has been suggested to promote LPS-induced endotoxin shock in mice by changing the balance between IL-12 and IL-10 production in favor of IL-12^35^. However, in contrast, others have shown that IDO1 mediates protection of mice from endotoxin shock in LPS-conditioned DCs^21^. More recently, TRP metabolism and the transcription factor AhR have been shown to mediate tolerance to infection *in-vivo*^13^. Accordingly, the role of IDO pathway in human endotoxemia and more importantly endotoxin tolerance has been controversial and remains elusive.

Endotoxin tolerance is a phenomenon characterized by unresponsiveness to a second exposure to LPS following an initial encounter with endotoxin that affects most of the TLR4-expressing innate immune cells^17^,^18^,^19^,^20^. DCs are sentinels and sensors of the immune system linking innate with adaptive immunity by inducing T cell activation or tolerance^14^,^15^. In addition, DCs are the major producers of IDO in the immune system^3^,^4^. Here we investigated how the IDO pathway modulates DC phenotype and function under endotoxin tolerance conditions and the role of the NF-κB pathway in this process. We have shown that both IDO isoforms as well as IDO activity are induced at much higher levels in LPS-conditioned DCs compared with unprimed controls (Fig. 1A,B,C). In addition, we found a similar pattern for AhR expression as well as activity (Fig. 1D). Moreover, we found an increase in anti-inflammatory mediators including IL-10 and the inhibitory molecules PD-L1 and PD-L2 in LPS-conditioned DCs compared with single stimulated controls (Fig. 2A,B). Furthermore, our data show that this induction was partially dependent on IDO activity (Fig. 3A,B,C) and does not involve changes in the expression of the TLR4 receptor complex (Fig. 2C). Hence, these data suggest that prolonged LPS stimulation in DCs induces an anti-inflammatory phenotype in an IDO-dependent manner. However, additional experiments should be performed to better understand the kinetics and the nature of sub-cellular changes leading to such changes. Finally, given the relatively small suppression in PD-L1 expression, and to some extent IL-10 production, in the presence of MTHT it is reasonable to suggest that IDO only partially accounts for the phenotypical changes observed in DCs and as such we cannot rule out that additional mechanisms might be involved.

The differences observed in our model in terms of pro-inflammatory cytokines compared with previous studies in mouse DCs are most likely due to specie-specific characteristics, which have also been previously suggested by other authors^36^. In addition, differences in LPS concentrations can also account for the dissimilar readouts, which suggest that LPS-induced immunological tolerance of DCs is complex and tightly regulated; and influenced by different environmental factors. In line with this, we have also found for the first time an induction in IDO2 gene expression in LPS-conditioned human DCs, which is in contrast to what has been previously observed in mouse splenic DCs^21^. Even though the affinity of IDO1 for TRP is much higher than IDO2^37^, it has been previously suggested that IDO2, but not IDO1, is involved in the development of several conditions including arthritis^38^. This provides evidence of separate *in-vivo* functions for IDO1 and IDO2. Additionally, using IDO knockout models, it has been shown that IDO2 is critical for IDO1-mediated T cell regulation, which suggests a non-redundant function in inflammation^39^. Furthermore, it has been demonstrated that IDO2 supports the homeostatic generation of T regulatory cells by human DCs^40^. All these data suggest that IDO1 and IDO2 might have non-redundant roles in regulation of endotoxin tolerance in a human context. On the other hand, it is also plausible that the differences observed arise from cell specific characteristics. Splenic DCs are naturally occurring DCs, while monocyte-derived DCs are mainly found under inflammatory conditions. Accordingly, future studies should also aim at studying whether this phenotype is a particular feature of inflammatory DCs or is shared among various DC subtypes.

Mechanistically, it has been suggested that the transcription factor Twist-2, a negative regulator of the TLR4 pathway, can modulate the production of pro-inflammatory cytokines like IL-12, IL-6 and TNF-α under endotoxin tolerance conditions in mouse myeloid DCs^41^. In addition, a similar role for the TLR4 adaptor protein interleukin-1 receptor-associated kinase (IRAK)-1 has been suggested in human DCs^42^. The NF-κB pathway exhibits great plasticity in inflammatory TLR signaling and also plays a central role in the resolution of inflammation^43^. TLR4 signaling can induce both the canonical and non-canonical NF-κB pathway. The canonical pathway starts with IKKβ-dependent phosphorylation and proteosomal degradation of IκB resulting in the release of the p65-p50 dimers. Subsequent p65 phosphorylation and nuclear translocation leads to the induction of pro-inflammatory cytokine expression that activates the immune response. In contrast, the non-canonical pathway involves IKKα-mediated phosphorylation of p100 associated with RelB, which leads to p100 processing and the generation of the p52-RelB complexes^44^. Switch in NF-κB function from an inflammatory (MyD88-mediated) to an anti-inflammatory (TRIF-dependent) mode has been observed in the course of inflammation and in the context of endotoxin tolerance^17^,^20^,^45^. Overexpression of inhibitory p50 homodimers and decreased levels of transcriptional active p65-p50 heterodimers have been observed in endotoxin tolerant monocytes and macrophages^46^,^47^. In addition, p50^−/−^ murine macrophages fail to develop endotoxin tolerance^36^,^48^. Members of the non-canonical pathway have also been implicated in endotoxin tolerance. Particularly, overexpression of RelB has been found in endotoxin tolerant human monocytes^49^,^50^. Accordingly, we focused our attention in studying the role of both arms of the NF-κB pathway in LPS-induced tolerogenic phenotype in human DCs. We found an induction in the non-canonical NF-κB pathway in LPS-conditioned DCs after TLR4 engagement. Overexpression of RelB along with NIK protein as well as down-regulation in TRAF3 and phospho-p65 were found in LPS-conditioned DCs compared with unprimed controls.

Finally, we further investigated the link between RelB’s regulation and the IDO pathway after LPS conditioning. Our data clearly showed that RelB expression was dependent on the IDO pathway (Fig. 5D). In particular, we found a significant downregulation in RelB expression in LPS conditioned DCs expressing low levels of AhR after gene silencing (AhR^low^-DCs) (Fig. 5A,B,C) compared to CT-DCs (Fig. 5D). Furthermore, we also found that RelB levels were recovered in the presence of IDO metabolite KYN. These data show a link between the IDO pathway and RelB modulation under endotoxin tolerance conditions and provide new insight into the mechanisms of LPS-induced tolerogenic phenotype of human DCs and endotoxin tolerance.

The hygiene hypothesis, which describes the protective influence of repeated microbial exposure in early life on the development of allergy and asthma, can be described as an induced tolerogenic state to exaggerated inflammatory response to allergens^51^. This phenomenon of tolerance to allergens can be controlled by mechanisms similar to those induced in an endotoxin tolerance state. Therefore, it could be interesting to explore the role that the IDO pathway might potentially play in protecting against developing allergic responses^7^. In this context, we have previously suggested that a functional and physical association between AhR and RelB might be modulating IDO levels under allergic responses^7^. Experiments are under way to demonstrate such associations. This could also have implications in endotoxin tolerance that will remain to be established.

In conclusion, these data highlight the role of IDO in LPS-induced immunological tolerance. LPS-conditioning in human DCs induces gene re-programming with induction of anti-inflammatory mediators which is also associated with an induction of the non-canonical NF-κB member RelB in an IDO-dependent manner. Future studies should focus on characterization of the epigenetic and transcriptional signatures of these phenomena. These data can help to further understand the mechanisms behind LPS modulation of DC phenotype and function under steady state and inflammatory conditions.

## Materials and Methods

### Generation of human DCs

Buffy coat samples were obtained from healthy volunteers in accordance with the relevant guidelines after obtaining informed written consent and approval of local ethics committee (all approved by the National Blood Service, UK). The methods were performed in accordance with the approved guidelines and regulations. DCs were generated as described before^52^,^53^. Briefly, peripheral blood mononuclear cells were separated by density gradient centrifugation on Histopaque (Sigma-Aldrich). CD14^+^ monocytes were isolated from PBMCs by positive selection using a magnetic cell separation system (Miltenyi Biotech, UK) (purity >95%) and were then seeded in RPMI 1640 medium (Sigma-Aldrich) supplemented with heat inactivated Foetal Bovine Serum (10%v/v), penicillin (100 U/ml) - streptomycin (100 μg/ml) and L-Glutamine (2 mM) (all from Sigma-Aldrich). Monocyte differentiation into DCs was carried out for 6 days in the presence of GM-CSF (50 ng/ml) and IL-4 (250 U/ml) (both from Miltenyi Biotech). The purity of the DC population was always ≥95%.

### Quantification of IDO activity

This was done as we have previously described^7^,^53^. Briefly, DCs were seeded in a 24-well plate with complete media supplemented with L-TRP (100 μM) (Sigma-Aldrich). For LPS priming, cells were stimulated with LPS (0.01 μg/ml, Sigma) once at the beginning of experiments and again after 24 hrs in the culture. Upon the second LPS stimulation, cultures were continued for a further 24 hrs. Control cells were left untreated for the initial 24 hrs and then were stimulated with same dose of LPS for the last 24 hrs.

After stimulation, IDO activity was measured by quantification of the levels of kynurenine (KYN) in the culture supernatant using a colorimetric assay as we have described previously^7^,^53^. IDO inhibitor methyl-thiohydantoin tryptophan (MTHT) (100 μM) and its vehicle DMSO as well as the IDO metabolite KYN (50 μM) were purchased from Sigma-Aldrich.

### RNA isolation, cDNA synthesis and qPCR

For LPS priming, cells were stimulated or not with LPS (0.01 μg/ml, Sigma) for 24 hrs and then received the same dose of LPS for different time points. Cells were washed twice in ice-cold PBS and total RNA extraction was carried out using the RNeasy Plus Minikit (Qiagen, UK) according to manufacturer’s instructions. Samples were then DNase treated using the TURBO DNA-free kit (Thermo Fisher Scientific) and total RNA was concentrated through ethanol precipitation. cDNA was synthesized from total RNA using superscript III first-strand synthesis kit (Thermo Fisher Scientific) according to manufacturer’s instructions. Quantitative real time PCR was performed in a Strategene MxPro 3005 P qPCR System with the Brilliant III Ultra-Fast SYBR Green qPCR Master Mix (Agilent Technologies, USA). Cycling was initiated at 95 °C for 3 min, followed by 40 cycles of 95 °C for 20 secs and 60 °C for 20 secs, a melting curve was done at the end. Samples were run in triplicates, and relative expression was calculated using the comparative threshold cycle method, also known as the ΔΔCt method, normalized to GAPDH or 18S rRNA^54^. Ct values from GAPDH and 18S rRNA did not change by more than 0.5 to 1 cycle from sample to sample. Primers were obtained from Eurofin, UK: Glyceraldehyde 3-phosphate dehydrogenase (GAPDH) Forward (5′-GAGTCAACGGATTTGGTCGT-3′), GAPDH Reverse (5′-GACAAGCTTCCCGTTCTCAG-3′), 18S rRNA Forward (5′-GTAACCCGTTGAACCCCATT-3′), 18S rRNA Reverse (5′-CCATCCAATCGGTAGTAGCG-3′), IDO1 Forward (5′-GGCACACGCTATGGAAAACT-3′), IDO1 Reverse (5′-GAAGCTGGCCAGACTCTATGA-3′), IDO2 Forward (5′-CTGATCACTGCTTAACGGCA-3′), IDO2 Reverse (5′-TGCCACCAACTCAACACATT-3′), AhR Forward (5′-ATCACCTACGCCAGTCGCAAG-3′), AhR Reverse (5′-AGGCTAGCCAAACGGTCCAAC-3′), CYP1A1 Forward (5′-CACAGACAGCCTGATTGAGCA-3′), CYP1A1 Reverse (5′-GTGTCAAACCCAGCTCCAAAGA-3′), RelB Forward (5′-TCGTCGATGATCTCCAATTCAT-3′) and RelB Reverse (5′-CCCCGACCTCTCCTCACTCT-3′).

### Flow cytometry analysis

Antibodies (Abs) against CD80 (clone MAB104), CD83 (clone HB15a), CD86 (clone HA5.2B7), MHC-II (clone Immu-357) and DC-SIGN (clone AZND1) were purchased from Beckman coulter, UK. Abs against PDL2 (clone MIH18), PDL1 (clone MIH1) and ICOSL (clone 2D3/B7-H2) were purchased from BD Biosciences. Abs against CD14 (clone TUK4) and CD40 (clone HB14) were purchased from Miltenyi Biotech. Ab against AhR (clone FF3399) was purchased from eBioscience, UK. Ab against MR (clone 19.2) was purchased from Biolegend, UK. Ab against TLR4 (clone HTA125) was purchased from AbD serotec, UK. Ab against MD2 (clone 18H10) was purchased from abcam, UK. Nonreactive isotype-matched Abs were used as controls. Cell staining and flow cytometry analysis was performed as described before^7^,^55^. Briefly, cells were harvested and washed twice with PBA (PBS containing 0.5% BSA and 0.1% sodium azide) (all from Sigma-Aldrich). At this point surface staining was done for 20 min at 4 °C. For intracellular staining, cells were fixed for 10 min at room temperature with formaldehyde (4%) (Sigma-Aldrich). They were then washed twice with permeabilization/wash buffer (PBA containing 0.5% saponin (Sigma-Aldrich)), stained for 30 min at 4 °C and washed twice in the same buffer before analysis in an FC 500 Flow Cytometer (Beckman Coulter)^7^. All data analysis was done using Weasel Software.

### Cytokine measurements

Cell-free supernatants were collected and stored at −20 °C before analysis. Cytokine (IL-10, IL-12p70, IL-6, IL-8, TNF-α and IL-1β) concentration was analysed using the ProcartaPlex Multiplex Immunoassay system (eBioscience) according to manufacturer’s instructions. IL-10 levels were also measured using the DuoSet ELISA System (R&D Systems) according to manufacturer’s instructions.

### Reverse phase protein microarray

After stimulation, DCs were washed twice with ice-cold PBS and lysed in 60 μl of RIPA buffer containing protease and phosphatase inhibitors (all from Thermo Fisher Scientific). For reverse phase protein microarray the procedure described in Negm *et al*. was followed^26^. After denaturation, samples were spotted onto nitrocellulose-coated glass slides (Grace Bio-labs) with a microarray robot (MicroGrid 610, Digilab). Then, slides were incubated overnight in blocking solution (0.2% I-Block (Thermo Fisher Scientific), Tween-20 (0.1%) in PBS (Sigma-Aldrich) at 4 °C. After washing, slides were incubated with primary Abs overnight at 4 °C. β-actin Ab was included as a loading control. All Abs were purchased from Cell Signaling Technologies. After washing, slides were incubated with infrared Licor secondary abs for 30 min at room temperature in the dark. Finally, slides were scanned with a Licor Odyssey scanner (LI-COR, Biosciences). The resultant TIFF images were processed with Genepix Pro-6 Microarray Image Analysis software (Molecular Devices Inc.). Protein signals were determined with background subtraction and normalization to the internal housekeeping targets. Signal values represented on the colour scale for the heat map are log2 transformed from the arbitrary fluorescence units (AFU) and normalized by using the standard deviation. All fluorescent signals are reported as AFU with β-actin normalisation. Heat maps were generated using TMEV software.

### RNA interference

Small interfering RNA (siRNA) was carried out as previously described with slight modifications^7^,^56^. AhR siRNA was the SMARTpool: ON-TARGETplus siRNA from GE Healthcare, UK. The control siRNA was the ON-TARGETplus Non-targeting control from GE Healthcare. Monocytes were transfected on day 0 with either the specific AhR siRNA or a control scrambled (non-specific siRNA) (50 nM) using the HiPerFect Transfection Reagent. The inhibition of target gene was assessed at day 6.

### Statistical analysis

Values shown for each data point are the mean ± SEM from independent experiments using different donors unless otherwise stated. Data were assumed to follow a normal distribution and homogeneity of variance. Student t Test (to compare two groups) or ANOVA with Tukey’s post hoc Test (to compare three of more groups) were used to determine statistical significance. *p ≤ 0.05; **p ≤ 0.01; ***p ≤ 0.001; ****p ≤ 0.0001. For all statistical analysis GraphPad Prism 5 software (GraphPad Software, La Jolla, USA) was used.

## Additional Information

**How to cite this article**: Salazar, F. *et al*. The role of indoleamine 2,3-dioxygenase-aryl hydrocarbon receptor pathway in the TLR4-induced tolerogenic phenotype in human DCs. *Sci. Rep.* **7**, 43337; doi: 10.1038/srep43337 (2017).

**Publisher's note:** Springer Nature remains neutral with regard to jurisdictional claims in published maps and institutional affiliations.
